# Object Recognition in Flight: How Do Bees Distinguish between 3D Shapes?

**DOI:** 10.1371/journal.pone.0147106

**Published:** 2016-02-17

**Authors:** Annette Werner, Wolfgang Stürzl, Johannes Zanker

**Affiliations:** 1 Institute for Ophthalmic Research, Centre for Ophthalmology, Eberhard-Karls-Universität, Tuebingen, Germany; 2 Institute of Robotics and Mechatronics, German Aerospace Center (DLR), Wessling, Germany; 3 Department of Psychology, Royal Holloway University of London, Egham, United Kingdom; University of Sussex, UNITED KINGDOM

## Abstract

Honeybees (*Apis mellifera*) discriminate multiple object features such as colour, pattern and 2D shape, but it remains unknown whether and how bees recover three-dimensional shape. Here we show that bees can recognize objects by their three-dimensional form, whereby they employ an active strategy to uncover the depth profiles. We trained individual, free flying honeybees to collect sugar water from small three-dimensional objects made of styrofoam (sphere, cylinder, cuboids) or folded paper (convex, concave, planar) and found that bees can easily discriminate between these stimuli. We also tested possible strategies employed by the bees to uncover the depth profiles. For the card stimuli, we excluded overall shape and pictorial features (shading, texture gradients) as cues for discrimination. Lacking sufficient stereo vision, bees are known to use speed gradients in optic flow to detect edges; could the bees apply this strategy also to recover the fine details of a surface depth profile? Analysing the bees’ flight tracks in front of the stimuli revealed specific combinations of flight maneuvers (lateral translations in combination with yaw rotations), which are particularly suitable to extract depth cues from motion parallax. We modelled the generated optic flow and found characteristic patterns of angular displacement corresponding to the depth profiles of our stimuli: optic flow patterns from pure translations successfully recovered depth relations from the *magnitude* of angular displacements, additional rotation provided robust depth information based on the *direction* of the displacements; thus, the bees flight maneuvers may reflect an optimized visuo-motor strategy to extract depth structure from motion signals. The robustness and simplicity of this strategy offers an efficient solution for 3D-object-recognition without stereo vision, and could be employed by other flying insects, or mobile robots.

## Introduction

Insects show a remarkable complexity of visual performances, in particular in consideration of their comparatively small nervous system. For example, bees use the polarisation pattern of skylight and the analysis of optic flow fields from both eyes to navigate safely through their environment (reviewed in [1, 2]) and are amongst the best studied invertebrate models for colour and pattern vision [3, 4, 5, 6, 7, 8]. Also remarkable are the bees’ highly developed colour vision (exhibiting for example colour constancy [6, 7]) and their ability to recognize and generalize complex visual patterns (e.g. [8]).

In contrast, surprisingly little is known about whether and if so, how bees perceive the three-dimensional form of objects. To recover depth from the 2D retinal image is a major challenge for any visual system and humans exploit a multitude of sources, such as ocular information (position of the eyes, accommodation), pictorial cues (e.g. texture gradients, shadows), stereopsis and dynamic information from the optic flow field (e.g. motion parallax). Most insects, on the other hand, are thought to have a limited access to the three-dimensional aspects of the world because of the fixed optics of the compound eyes and limited binocular field of view. Instead, bees and other insects rely for their navigation in space predominantly on the analysis of the optic flow field [9–17]. The optic flow field is a distribution of local speed (magnitude) and direction of the image points on the retina as the observer moves. Surfaces in different depth planes will produce motion parallax, the shift of image points relative to each another. It has been shown previously that bees use motion parallax to estimate the distance to a target [10, 15] and to detect small targets [18] or otherwise camouflaged shapes against a patterned background, e.g. flowers in scenes mottled with shadows [19–22]. But can bees employ the same strategy also for encoding the three-dimensional structure of objects? To find out, we conducted behavioural experiments whereby we tested the bees ability to discriminate between three-dimensional solid objects. Using folded cards and photographic images of these stimuli we examined possible cues and strategies the bees might use for encoding 3D information, i.e. global shape, perspective cues and/or specific optic flow patterns.

## Material and Methods

### Subjects and Procedure

**Fig 1:**
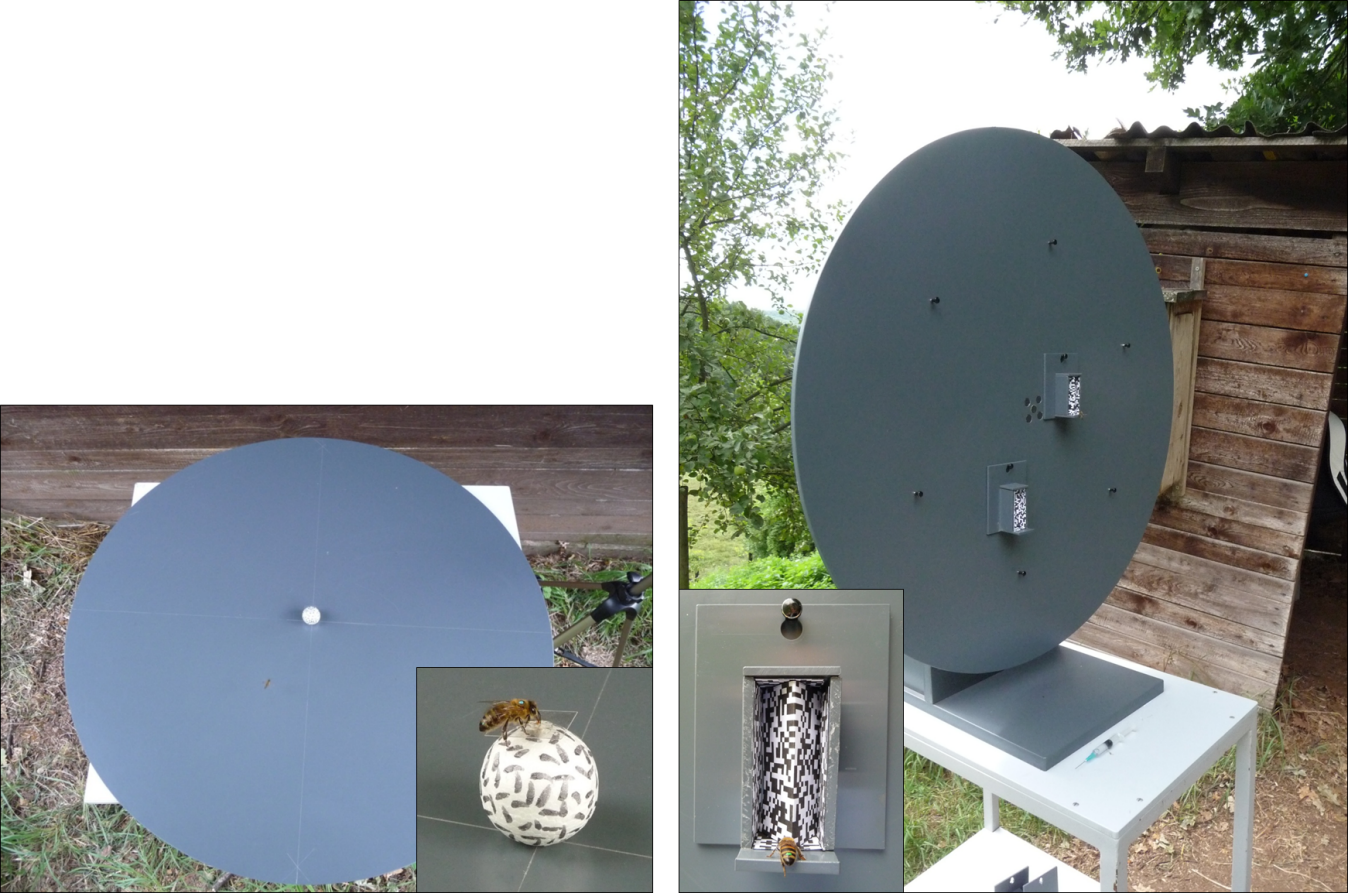
Experimental set up and examples for training bees on 3D stimuli. (a) Photo on the left shows set up used to test discrimination of solid objects (experiment 1); (b) photo on the right shows set up used to test discrimination of card stimuli (experiment 2); small inserted figures show bees rewarded on a ball stimulus (left), or in front of a convex card (right) during the initial training.

Individual, free flying honey bees (A*pis mellifera*, n = 46) from a nearby bee hive were trained to visit the experimental set up, which was situated outdoors in an open meadow. Two different experimental set ups (Fig 1) were used, which are described in detail in the corresponding sections below. The experiments were conducted in the shade of an umbrella, which shielded the apparatus and stimuli from direct skylight.

The experiments commenced with a one day training period; a group of two to three individually marked bees was trained to visit the set up, whereby only the training stimulus was presented; each visit to the training stimulus was rewarded with a drop of sugar water from a syringe (Fig 1, small inserts). For each of the experiments, a fresh group of bees was trained. Of the trained bees, only one at a time participated in the following testing, the remaining bees were meanwhile kept in a small cage and released to participate in later tests.

After one day of exclusively training, the actual experiments commenced. Each experiment started with 20 training visits (initial training), and was organized in sessions, each consisting of alternating testing and re-training periods. The initial training, as well as the re-training between the tests was carried out as differential conditioning, i.e. rewarded and unrewarded stimuli were presented simultaneously. No reward was given during the tests. Each test lasted 2 min, and was immediately followed by re-training the bees for 15 min, which allowed 3–4 visits of the bees. In order to avoid that the bees used odour marks, different sets of stimuli were used during training and testing and the background surface was cleaned with 90% ethanol.

The experimental conditions were presented in pseudo-randomized order. Discrimination was tested in a two-alternative-choice paradigm, whereby the training stimulus and one of the alternative stimuli were presented simultaneously (discrimination test). During the test period, the choice behaviour of the test-bees was recorded. Landing or briefly touching the stimulus was counted as a decision for that particular stimulus.

### Experimental setup and stimuli

Two sets of experiments were conducted, testing the discrimination of geometric objects or folded cards, respectively:

#### Experiment 1: geometric 3D objects

The set up consisted of a horizontal round turntable (plastic material, diameter 800 mm, grey to the human eye; Fig 1), which was placed on a stand (height 500 mm). The actual stimuli (Fig 1) were three-dimensional objects made of white styrofoam; their surface was covered with a random pattern of black oblong patches (1 x 3 mm). The following objects were presented: a sphere (diameter 30 mm), a cube (30 x 30 mm), a cylinder (30 x 5 mm) and a cuboid (30 x 30 x 5 mm). In one experiment, the height of cuboid/cylinder was raised by a thin stick (total height 30 mm). During training, the rewarding sugar water was presented on a cover glass (10 x 10 mm, see Fig 1A, small insert), positioned on top of the training stimulus. No cover glasses were present during the tests. In the initial training phase (first 5 visits), only the rewarded stimulus was present; training before and in-between tests was carried out as differential training, i.e., the respective alternative stimulus (to be tested in the following test interval) was also present.

#### Experiment 2: folded and planar cards

In experiment 2, the set up consisted of a vertical round turntable (made of the same material as the horizontal plate; diameter 800 mm; Fig 1B), which was placed on a stand (height 800 mm), facing north. The stimuli were positioned in small boxes (25 x 25 x 60 mm), made of the same material as the turntable and presented on one of eight positions on the turntable. Horizontal landing-boards (width 5 mm) attached to the front of the box, carried the rewarding drop of sugar water (positioned at the edge of the landing board) during the training sessions.

The actual stimuli were made from cardboard and were either planar (25 x 60 mm) or folded (50 x 60 mm) along the vertical midline in one of two configurations, concave or convex (opening angle α = 60°; see Fig 1B). The cards, as well as all inner sides of the stimulus boxes, were covered with a black & white random block pattern (3 x 3 mm per block). This should provide visual cues for texture gradients, and motion stimuli. In addition to these “real” stimuli, also black & white photographs of the stimuli were presented. The photos (Panasonic DMC-TZ5) were printed on paper (1:1 scale). The printouts were positioned within the boxes, in the same plane as the “real” planar stimulus, i.e. at a distance of 12.5 mm from the front edge (the stimulus midline). In the initial training phase (first 5 visits), only the rewarded stimulus was present; training before and in-between tests was carried out as differential training, i.e., the two alternative stimuli were also present.

#### Statistical Analysis

We used a two-tailed *t*-test to evaluate the difference of the number of decisions for the training and the unrewarded (alternative) stimuli, respectively. A 2 x 2 contingency table and the Pearson *χ*^*2*^ test were used in order to assess the significance of the difference of the test-results involving the ball as the training stimulus and the cube as the training stimulus, respectively.

## Results

### Experiment 1: Discrimination of geometric objects

**Fig 2:**
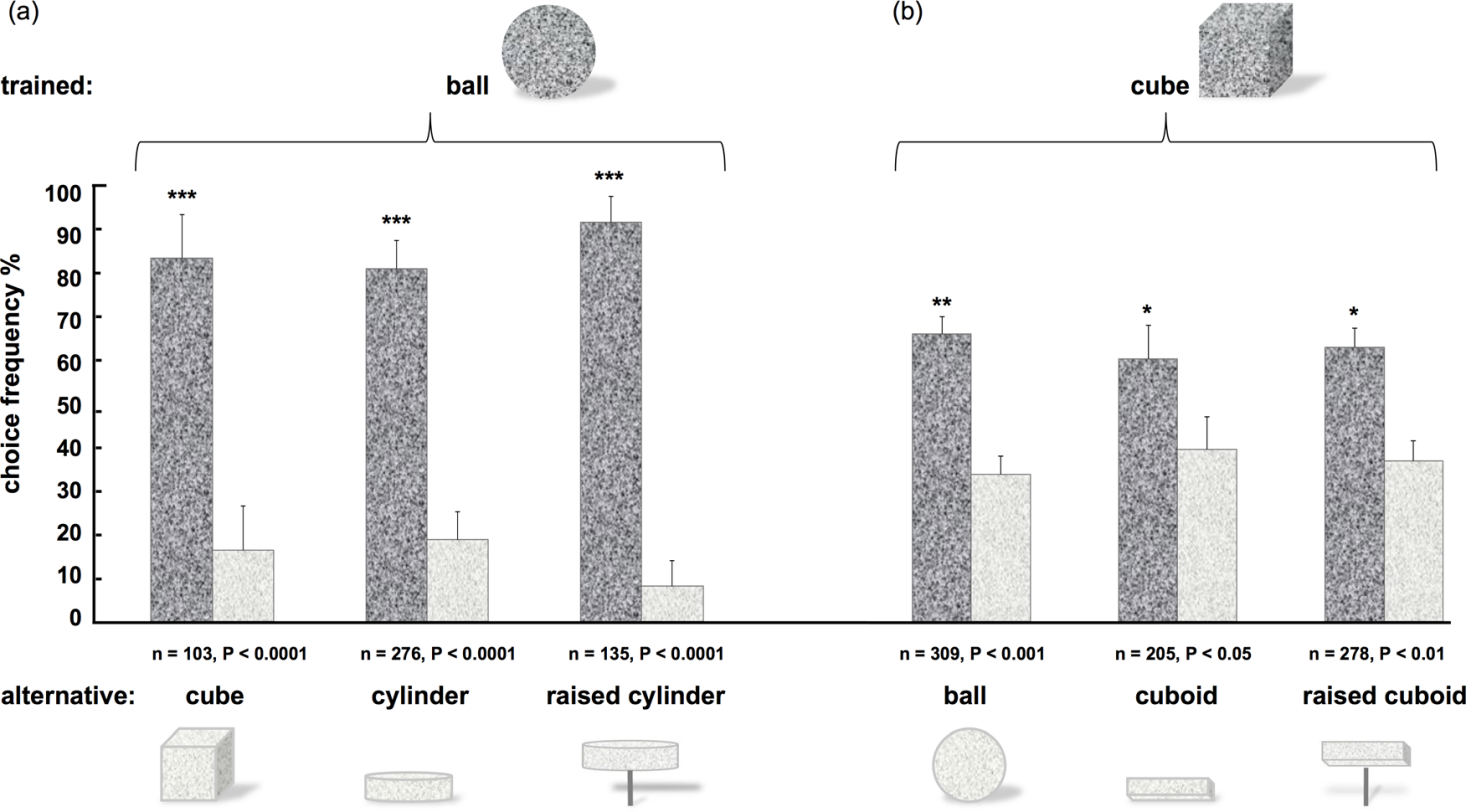
Discrimination between solid objects (experiment 1). (a) trained stimulus: ball; alternative stimuli: cube, cylinder, raised cylinder. (b) trained stimulus: cube; alternative stimuli: ball, cuboid, raised cuboid. In each case, dark bars indicate the bees proportion of choices for the trained stimulus, light bars indicate the proportion of choices for the respective alternative. Stimuli were tested pair-wise, asterisks denote significant discrimination (*t*-test), error bars indicate standard error of the mean; n is the total number of decisions made in each experiment, respectively.

In the first set of experiments, individually marked honey bees (*Apis mellifera*) were trained to discriminate between geometric objects such as a ball, a flat cylinder, a cube, and a cuboid. Training stimuli were a ball (in the first experiment) and a cube (in the second experiment), whereby the training object and one of the alternative, unrewarded objects (which was to be discriminated in the following tests) were placed on the horizontal round table (Fig 1A), at equal distance from the centre. The results are shown in Fig 2 and reveal an excellent discrimination of the objects (Fig 2A: ball vs. cube: P < 0.0001; Fig 2B: cube vs. ball: P < 0.001).

This result may be surprising given the reported difficulties of bees to discriminate simple geometric 2D shapes when presented horizontally, for example squares vs. triangles [23–25]; however, it is known that the same 2D forms are easily discriminated when presented vertically [26, 21]; given that 3D objects contain additional vertical as well as horizontal components, this would predict a better discrimination of 3D stimuli. The failure to recognize 2D shapes when presented horizontally has been attributed to the variance of their retinal images with the bees viewing direction when approaching these stimuli. This variance of the retinal image may also explain the significantly (*χ*^*2*^, P < 0.001) lower discrimination values after training the cube as compared to training the ball: the outline of a cube is different from different viewing directions, whereas the retinal image of a ball is view-point invariant.

The presence or absence (in the case of the ball) or presence (in the case of the cube) of vertices and their number are strong cues for discriminating between different shapes [27, 28]. In order to see whether the bees discriminated the objects based on the absence or presence of corners, we tested discrimination of the ball and the cube against their flattened versions, i.e. a flat cylinder and a cuboid, respectively. As shown in Fig 2A and 2B, the bees preferred the trained stimuli in both cases, indicating that recognition did not depend on the detection of vertices alone (ball vs. cylinder: P < 0.0001; cube vs. cuboid: P < 0.05). We also excluded relative height of the surfaces as a cue for discrimination [10] by presenting the flat cylinder and cuboid on sticks, raised to the same height as the ball and the cube. Again, the bees had no difficulty to discriminate between the trained and alternative stimuli (ball vs. raised cylinder: P < 0.0001; cube vs. raised cuboid: P < 0.01), respectively.

Taken together, the first experiment showed that bees successfully discriminate 3D objects, independently of their height, or the presence of distinct shape features such as vertices. But which properties of the objects did the bees use for discrimination? In the case of two-dimensional stimuli, it has been suggested that bees encode, in addition to overall colour and texture, the global shape, predominantly by detecting the outer edges [29]. In fact, Lehrer and Campan [21] demonstrated that bees can learn the shape of geometric figures based on detecting their edges. We asked therefore: do bees rely on the outline shape (silhouette) for the discrimination of 3D objects? We tested this hypothesis explicitly in the following experiments.

### Experiment 2: Discrimination of planar, concave and convex cards

In the second set of experiments, we designed the stimuli so that they could not be discriminated based on their outer shape. To that aim, convex, concave or planar paper cards were positioned in small boxes (Fig 1B), and presented on the vertical turntable. Thus, the global shape (outer form) of all stimuli was identical and they differed exclusively in the depth profile of their frontal surface. Therefore, if bees relied for discrimination of the 3D objects exclusively on their outline shape, they should now confuse the different card-stimuli. If, however, they can extract the information about the inner depth profile of the stimuli, they should be able to distinguish plane, concave and convex card-stimuli.

**Fig 3:**
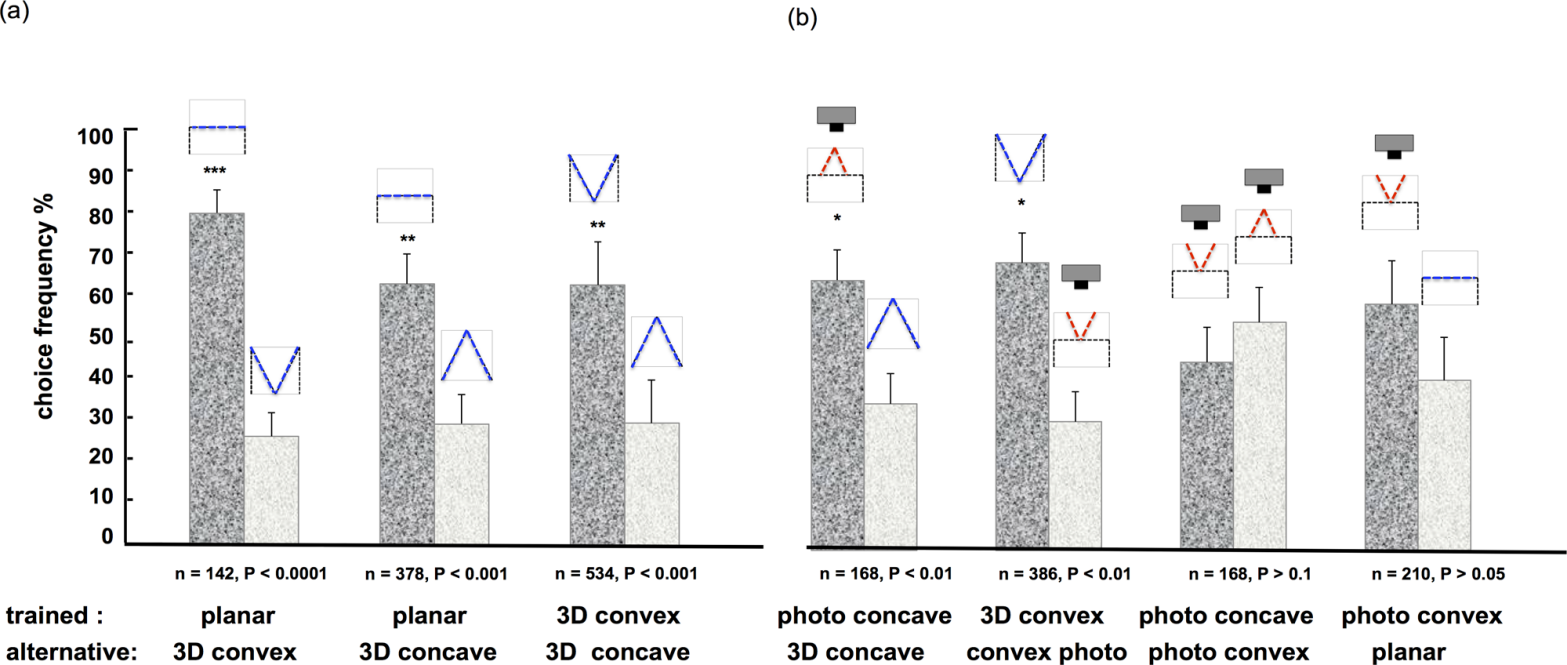
Discrimination of folded cards, planar cards and their photographic images (experiment 2). (a) discrimination between planar, 3D convex and 3D concave cards, sketches on top of each column represent the depth profiles of the corresponding stimuli, with black lines indicating the outline of the stimulus box, and blue lines marking the depth profile of the cards; (b) discrimination between folded cards (3D concave, 3D convex) and their photos (photo concave, photo convex) or the planar stimulus; photos are denoted also by the camera symbol in the top row, the red lines indicate the photographed depth profiles. Bees discriminate well between different 3D stimuli and between 3D stimuli and their corresponding photos; bees do not discriminate between the different photographic and/or planar stimuli. Stimuli were tested pair-wise, asterisks denote significant discrimination (*t*-test), error bars indicate standard error of the mean; n gives total number of decisions in each experiment, respectively.

The results are shown in Fig 3A. It can be seen that the bees safely discriminated between all the different spatial configurations, i.e. convex versus concave card (P < 0.001), planar versus concave card (P < 0.0001) and convex card versus concave card (P < 0.0001). This indicates that in these experiments, the bees used cues other than the outline shape (silhouette) for discrimination, in other words, they had access to the depth profiles of the stimuli.

In the second part of the experiments, we examined which depth cues the bees may use. Perspective cues from texture gradients or shading are powerful cues for depth perception in humans. So, given the bee’s acute pattern recognition [30, 8], could they employ similar strategies? In order to find out we produced photos of the “real”, three-dimensional stimuli (in the following denoted by the prefix “photo” and “3D”, respectively), which were then printed on planar cards and positioned in the stimulus-boxes. When trained to the “real” 3D stimulus, the bees had no difficulty in distinguishing it from the corresponding photo and vice versa (Fig 3B; photo concave versus 3D concave: P < 0.01; 3D convex versus photo convex: P < 0.01). Importantly, bees did not discriminate between the photographic images, namely, a photo of the concave stimulus versus a photo of the convex stimulus (P > 0.1) or a photo of the convex versus the planar stimulus (P > 0.05). This shows that the bees did not rely on texture gradients or shadows for the discrimination. So, what information did they use instead?

A conceivable candidate would be information from the optic flow field; for example, during a lateral movement of the bee, the speed of an image shift on the bees retina from the near sides of the card is larger than the speed from the inner fold of the card which is further away and therefore indicates a concavity. In contrast, a planar card would produce a rather homogenous flow profile across its surface. In order to find out whether the bees could use signals from motion parallax to uncover the depth profiles of the cards, we set up high speed cameras to film the bees during testing and modeled the optic flow patterns obtained during these flights.

### Analysis of the flight behaviour of the bees in front of the card stimuli

**Fig 4:**
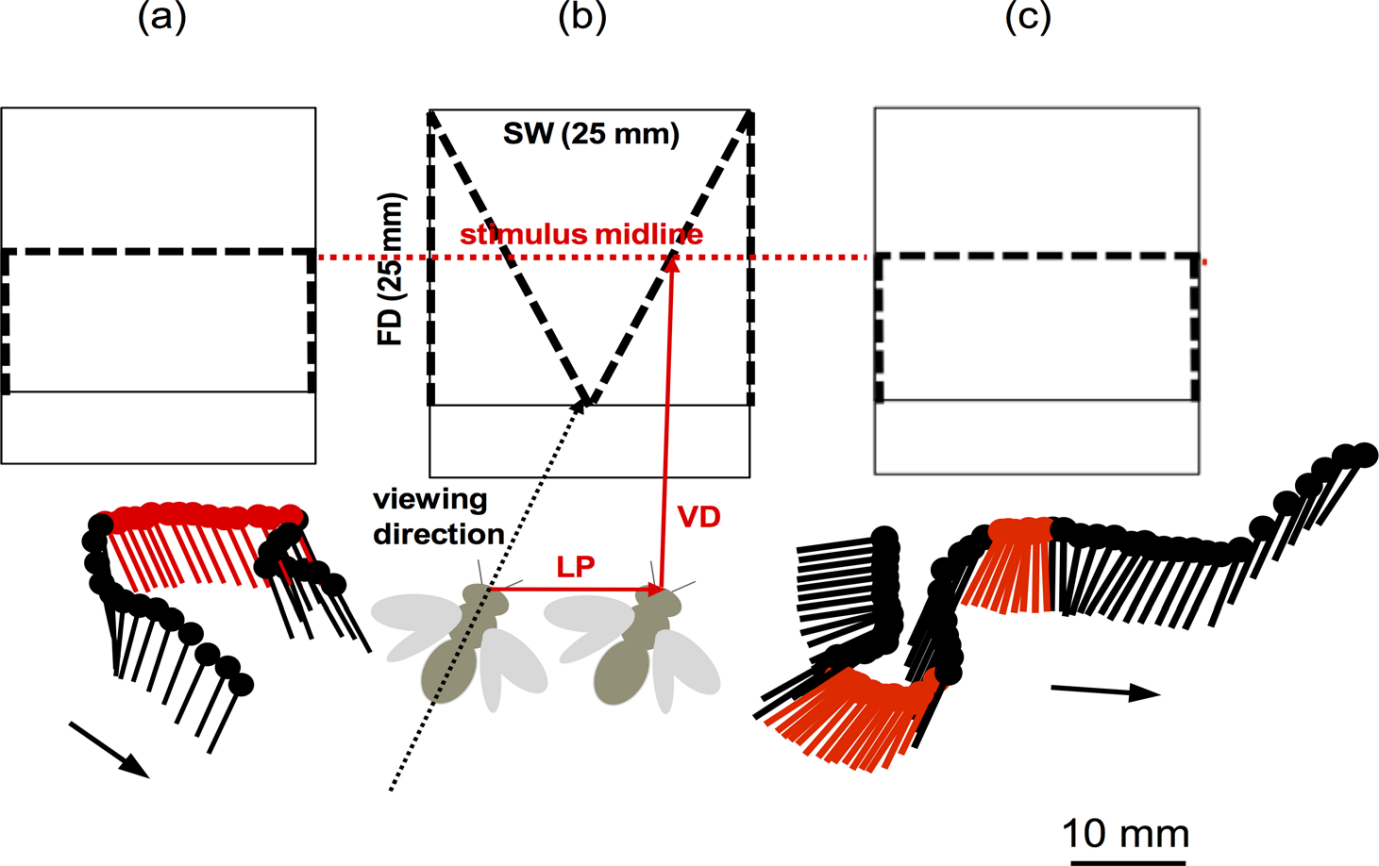
Schematic sketch of the stimuli and flight tracks of the bees. Top row: schematic sketch of the stimuli, as seen from above; solid lines indicate the outlines of the stimulus boxes, overall stimulus width (SW) and field depth (FD) 25 x 25 mm; broken black lines show depth profiles of the cards (planar (a, c), convex (b)); bottom row: (a, c) typical flight tracks of the bees, as seen from above, dots indicate head-position of the bee, arrows indicate direction of flight; translation (a, marked in red), translation + rotation (c, marked in red); (b) shows a schematic sketch of the geometric variables (VD refers to viewing distance, LD to lateral displacement, the thin black dotted line marks the bees’ body orientation).

Two high-speed video cameras (Optronis, at 125 Hz temporal resolution), positioned at right angles on top and the side of the turn table, recorded the flight paths of four individual bees while approaching and discriminating between the different card stimuli. Fig 4 (see also Figure A in S1 Text) shows typical episodes of flight tracks of a bee in front of a stimulus box, as filmed by the top camera, and the corresponding schematic sketches of the stimulus geometry: (1) a *translation*, whereby–within a fronto-parallel trajectory—the body axis is kept rather constant (“*pure translation*”, Fig 4A), at an angle of approximately 50 deg relative to the face plane of the stimulus and (2) a *rotation*, whereby the body axis rotated sideways (yaw) with an additional change in lateral position (“*translation + rotation*”, Fig 4C). Notably, 55% of the analysed flight maneuvers (n = 64) in front of the stimulus boxes included rotations, independently of the stimulus configuration (planar, convex or concave). As will be described below, this has an important consequence for the optic flow patterns generated in such flight trajectories: it is obvious from the stimulus geometry that a rotation component with the lateral translation will create a singularity in the optic flow without image shift [31, 32], which can stabilize one particular region of the stimulus on a fixed image point of the compound eye. In addition we also observed episodes of forward and backward movements which would induce a contraction and extension of the image, respectively, and could be used to normalise the distance to the stimulus [16].

### Modelling the motion parallax induced by the flight maneuvers

In the following we investigated whether the bees movements in front of the stimuli would have the potential to produce motion parallax patterns which reveal the depth profile of the cards. To this end, we first calculated the angular displacement generated for characteristic points on the folded stimulus cards in a horizontal plane defined by a purely lateral displacement and a cross-section through the stimulus, as an abstraction of the flight paths. Please note that these calculations are based on the idealized situation of a flight trajectory that is parallel to the front plane of the stimulus box and is constant in its height over ground; this is justified by the observation that, in this behavioural context, the bees maintained a largely constant height; furthermore, it should be noted that, although any additional variations in height and distance would change the absolute values of parallax shifts, this would not affect the overall pattern of results. We calculated the parallax shift measured in degrees of visual angle for an overall lateral positions shift of two sizes (LD = 2 or 16 mm) as seen from an average viewing distance VD = 27.5 mm.

**Fig 5:**
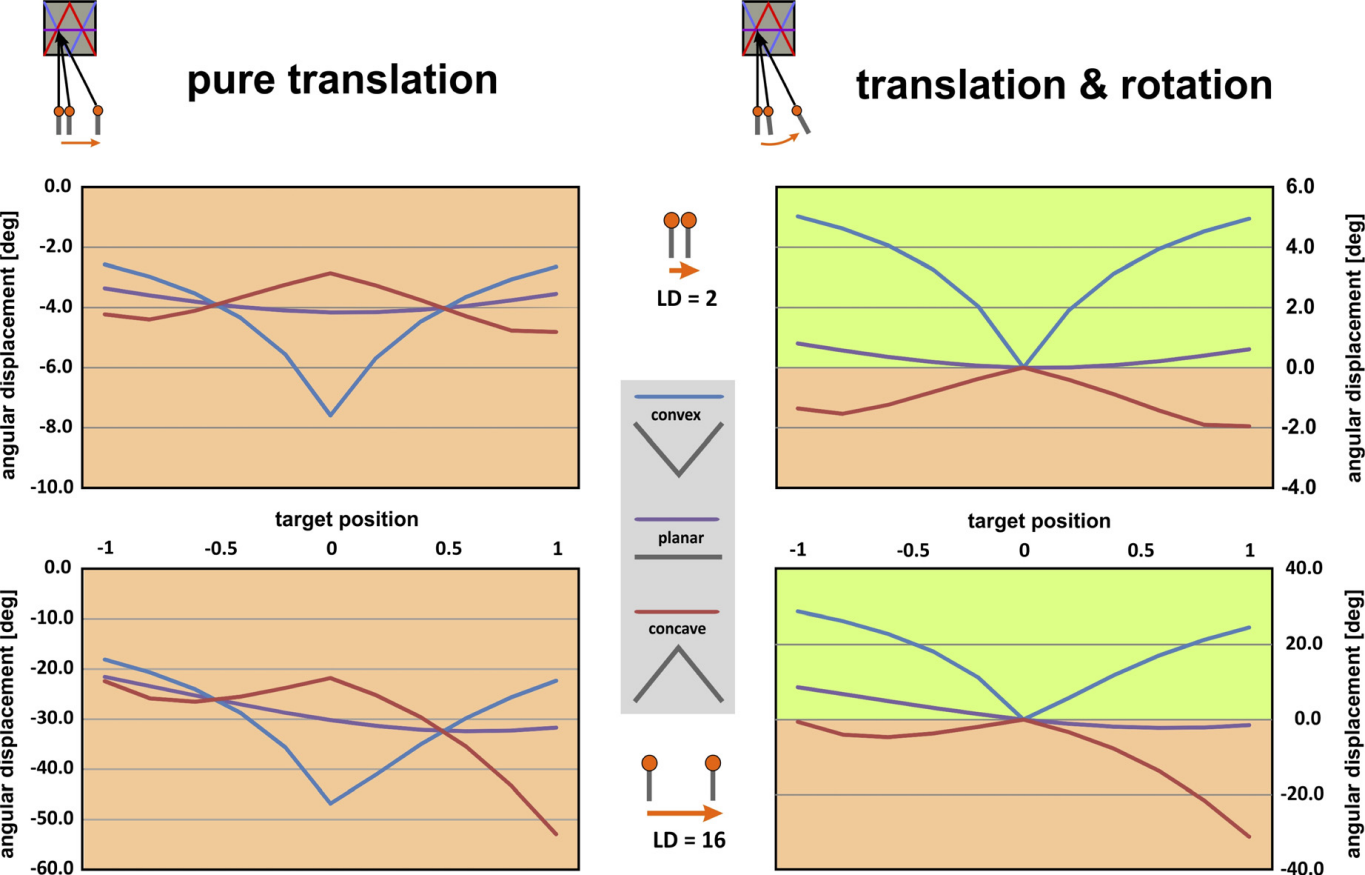
Optic flow patterns corresponding to the planar stimulus (purple line), the convex stimulus (blue) and the concave stimulus (red line). The four panels show patterns resulting from pure translations (left side), and combined translation and rotation (right side) and a small and a large lateral observer displacement (upper and lower panels, respectively). The bee’s flight path is assumed to be in a horizontal plane at a constant viewing distance of VD = 27.5 mm. Parallax shift (angular displacement) is plotted on the ordinate; pattern location on the stimulus (target position) is plotted on the abscissa, in relative units (-1 to +1 for an overall stimulus width of SW = 25 mm); the angular displacement values are calculated relative to the stimulus centre (position ‘0’) for the three configurations of the cards (convex, planar, concave), and two magnitudes of lateral observer displacements to the right (LD = 2, 16 mm). The light red regions indicate negative image shifts (to the left), whereas light green regions indicate positive image shifts (to the right). The response profiles are distinct and consistently negative for the three stimuli for pure translation (left panels), but are converted into generally positive displacements for convex, and negative displacements for concave stimuli, respectively, when the observer moves with translation + rotation (right panels). The asymmetry in the simulated displacement profiles is a consequence of the geometry of lateral translation and distance of the folded surface at close viewing distance: the angular size of the projected plane depends on its inclination, which amplifies angular displacement on one side and reduces it on the other side of the fold.

Image displacement by motion parallax was calculated with a geometric model for motion parallax, for a wide range of conditions. The outcome of the model is illustrated for a subset of typical conditions in Fig 5. Here, the angular shift of image points (in degrees of visual angle) for convex, planar and concave shapes across the full range of positions on the stimulus surface (scaled from -1 to 1 relative to the centre of the stimulus, position “0”) are shown for pure translation (left column), and translation + rotation (right column).

It can be seen immediately that the displacement profiles reflect the 3D shape of the stimulus cards in the amount of angular displacements relative to the midline of the stimulus. Notably, the magnitude of the image shift (about 4 deg for the planar stimulus, in the case of a lateral displacement of LD = 2 mm) and its speed (0.04 m/s for the translation), convert to an angular speed of 80 deg/s, which is clearly in a range that easily can be picked up by the bees’ visual system in order to extract such motion parallax profiles. In fact, slowing down in front of the stimuli, the bees flight speed is close to optimum for resolving moving gratings, i.e. 8–10 Hz [33]. Also, the observed minute size of the lateral displacements ensures a veridical response profile: when the observer displacement is increased (most obvious for the extreme case of LD = 16 mm), the magnitude of the parallax displacements is amplified correspondingly (lower graphs in Fig 5), but at the same time response profiles are distorted in the regions closer to the edges of the stimuli as a simple consequence of an increasingly asymmetric perspective of the stimulus as seen from a lateral viewpoint (i.e. as the bee moves from left to right). Note that the model predicts a more shallow profile of the angular displacement across stimulus width for the concave stimulus than for the convex stimulus; this is consistent with the behavioral results (Fig 3) showing a slightly better discrimination of the planar stimulus when tested against the convex stimulus (76%), as when tested against the concave stimulus (68%; see Fig 3). Taken together, the predictions of the model are in good agreement with the bees’ behaviour and discrimination performance.

Another observation may be worth pointing out: bees seem to find it harder to discriminate concave from convex stimuli than concave or convex from the planar stimulus. This correlates with the difference in complexity of the optic flow patterns of the planar stimulus on one hand and the concave and convex stimuli on the other; whereas the planar flow pattern has no abrupt discontinuities, the concave and convex map both have discontinuities; this would support the conclusion that bees identify the depth profile not only from velocity cues in the optic flow patterns but also by detecting features such as discontinuities at edges (see below).

Importantly, for pure translation, the parallax profiles for different 3D stimuli are always in the range of negative displacements (shown in orange in Fig 5), with a maximum/minimum amplitude in the stimulus centre; crossovers of the distinct profiles occur at the stimulus midline where points are located at the same distance from the observer’s eye in the horizontal image plane. In consequence, discrimination of the 3D configurations would depend on comparing relative motion *magnitudes* across space and thus on the bees’ accuracy to discriminate different speeds of the optic flow. For translation plus rotation, on the other hand, the parallax profiles for convex and concave stimuli are characterized by a positive and a negative sign of the displacement: this is because the lateral stimulus regions move behind or in front of the fixated and hence static image point (central part of the stimulus, i.e. closest point for convex, most distant point for concave stimuli), thus generating image displacements in opposite directions (positive values, indicated in green and negative values, indicated in red in Fig 5, respectively). Interestingly, the same sign inversion of parallax displacements has been described for orientation flights of solitary wasps [12, 34]. Thus, for the detection of 3D profiles, the bee does not need to rely on the discrimination of the speed of image points alone; instead, exploiting the sign (direction) of parallax shifts, provides a simpler and more reliable processing mechanism.

## Discussion

The results of our experiments demonstrated that bees possess an excellent perception of three-dimensional form: experiment 1 (Fig 2) showed that bees easily distinguish between different solid objects, whereby they could use either 2D or 3D cues or both. Furthermore, experiment 2 showed that bees also successfully identified the depth profile of surfaces (folded cards), independently of their global shape (Fig 3A). Presenting photographic images of the three-dimensional stimuli revealed that the bees did not rely for this task on pictorial cues, such as texture gradients or shadows (Fig 3B).

This is not to say that bees couldn’t—in general—use these features to uncover depth relations; for example, it has been observed in bumblebees that the detection of the orientation of stepped edges requires intensity information (Collett & Cartwright, pers. com.). Thus, bees could base their discrimination on differences in the form of shadows cast by the box on the different card configurations; however, different forms of shadows were also present in the photographic images, but were not used for discrimination, possibly because the shadow boarders were camouflaged by the high contrast random block pattern on the cards. Depth relations may also be revealed by the relative shift of the edges of the stimulus box and their shadow, as the bees move in front of it. However, since the photographic images, too, were placed *inside* the stimulus box, the same relative shift occurs, following the bees movements. We conclude therefore that, in our experiments, shadows would not provide suffient information for the bees to use as a cue to determine the depth profiles; instead our data suggest that bees use the information from motion parallax to extract the depth profiles of the stimuli.

### Motion parallax as a possible cue to depth perception in bees

Numerous studies have shown that bees analyse optic flow patterns for navigation tasks and to identify surfaces in different depth planes. Our results now indicate that bees deploy self-induced motion signals to accurately distinguish planar, convex and concave depth profiles. It is therefore interesting to examine the actual flight maneuvers of the bees in front of the stimuli.

Recordings of these flight paths revealed characteristic flight maneuvers which show fronto-parallel movement episodes with nearly constant body-axis (*translations*), but also, importantly, *rotations* of the body axis in a horizontal plane (yaw). This observation is important because by adding a rotational component to the translational parallax, the distribution of motion signals can be ‘anchored’ relative to a single point (e.g. the centre of the target) that is fixated by means of the rotation, whilst going through the lateral translation. In other words, it provides the static point in the optic flow field (see [32]). This will render flow patterns in which 3D profiles (convex or concave) are represented by the sign (positive or negative, respectively) of the motion signal map, in addition to different magnitudes (as provided by translation; see Fig 5). Therefore, the particular flight maneuvers (translations *and* rotations), which we observe in bees flying in front of the stimulus boxes, could reflect an optimized visio-motor strategy to extract the depth structure from optic flow.

It is noteworthy that the flight patterns in front of the stimuli are reminiscent of the so called “turn-back-and-look”behavior (TBL; [33]) or the translations and turns employed by wasps and bees in orientation flights, while they back away from the nest or a newly discovered food location [31]. In these cases, the insects turn and fly sideways along arc segments, centred around the goal, which in effect fixates the goal on their lateral eye region [34]. This behaviour is thought to be a stereotyped, innate flight pattern aimed at providing information about the spatial position of the goal. We also observed TBL in newly recruited bees during their first visits to the experimental set up. However, the flight maneuvers reported here for the bees differ from TBL, in that they were carried out upon arrival at the stimulus and throughout the duration of the tests, not when leaving the feeding place. Furthermore, the turns (rotations) were focusing on the inside of the stimuli, not the entire stimulus boxes (as in TBL, which was also carried out at a larger range) and therefore seemed to be directed at recovering the stimulus profile rather than the stimulus position. We conclude that the maneuvers of the bee when inspecting the stimuli during the tests may be related to, but are not identical, to TBL. Together, these observations suggest that the flight patterns of bees are highly adapted to provide several kinds of information: (1) navigation related information for estimating the position of a stimulus in space, possibly using the lateral eye regions; and (2) stimulus related information for identifying the 3D form of a stimulus, likely using the high-resolution of the frontal eye regions (for a review of other active vision strategies in bees see for example [35, 20]).

### How is 3D shape represented in the bees visual memory?

In general, bees could use a view-based strategy for object recognition [36]; that is, bees could construct a model of the 3D form of objects, such as the ball or the cube in experiment 1, from multiple 2D images, collected from different view points. Alternatively, or additionally, bees could use single or multiple features, and their spatial relations, to construct a 3D feature map, as proposed by structural models of human object recognition (see review in [37]). The latter strategy is supported by he finding that bees can discriminate complex patterns and shapes based on the presence or absence of learnt features in the actual stimulus [8, 21, 37–43]. The results from our experiments, in particular experiment 2, suggest that bees extract and store depth cues as distinct visual features of an object, together with its colour, texture and global shape [43].

The encoding of single object features, as compared to storing the entire visual image, can be understood as an economical strategy of particular importance for animals with small brains, because it would save visual memory. In fact, it has been suggested that the bees visual memory represents objects by a restricted set of features, namely the objects’ outline plus overall colour or texture, but sparing its internal structure [29]. Our findings extend this notion by showing that the inner (three-dimensional) structure of a stimulus is also encoded (see discrimination between planar, convex and concave cards). In keeping with a sparse visual code, we propose that depth profiles are stored in the bees’ visual memory by depth contour maps, similar to, but probably more detailed than, the optic flow maps used for recognizing landmarks [14, 17, 44].

## Conclusions

The experimental results showed that bees can successfully recognize the three-dimensional form of objects and surfaces, independent of pictorial cues or outer shape. We conclude that the bees employ a detailed analysis of optic flow and encode depth profiles in 3D-feature maps. For this performance, bees actively engage in specific flight maneuvers, which seem to be optimized for this task, namely a reduction of their speed of flight, and combinations of translations and rotations. We present a model suggesting that the optic flow generated by these flight maneuvers provides the information necessary to accurately identify the depth profiles. Thus, the depth features of objects can be encoded effectively in a tiny visual system, such as the honeybee’s visual system, using a simple and robust mechanism.

## Supporting Information

S1 Text Behaviour of the bees during discrimination tests. Figure A. Flight tracks of individual bees during discrimination tests.
(DOCX)
