# Supplementary material for: Object Recognition in Flight: How Do Bees Distinguish between 3D Shapes?
Source: PLoS One. 2016 Feb 17;11(2):e0147106. doi: 10.1371/journal.pone.0147106 (PMC4757030; doi:10.1371/journal.pone.0147106)
Supplement: S1 Text — Figure A. Flight tracks of individual bees during discrimination tests. (DOCX) [file pone.0147106.s001.docx]

**S1 Text. Behaviour of the bees during discrimination tests**

Figure A shows the flight tracks of individual bees during discrimination tests, as filmed by the top camera. A manual frame-by-frame analysis of 30 tracks, whereby we marked the bees’ head position and body axis, uncovered details of the bees behaviour. When approaching a stimulus, bees “inspected” it for several seconds, and then moved to the other stimulus, where they again engaged in their “inspection-flight”. The flight paths show a characteristic pattern of lateral excursions, with periods of constant body angle that are interspersed with periods during which the body axis is continually adjusted. Furthermore, it was found that bees reduced flight speed in front of the stimuli, from approximately 0.4 m/s when approaching the stimuli, to 0.04 m/s or less, when inspecting them. In contrast to newly recruited bees, which scanned the entire outline of the stimulus boxes, trained bees moved predominantly sidewise, in a horizontal plane, in front of the stimuli. Throughout these inspection flights, bees typically kept a roughly constant distance to the stimulus (26 – 28 mm; average of 25 approaches, measured orthogonally from the bees head to the stimulus midline), which we specify as the “viewing distance” VD (Fig 4 b). It is important to note that VD did not depend on the type of stimulus profile (planar, concave or convex).


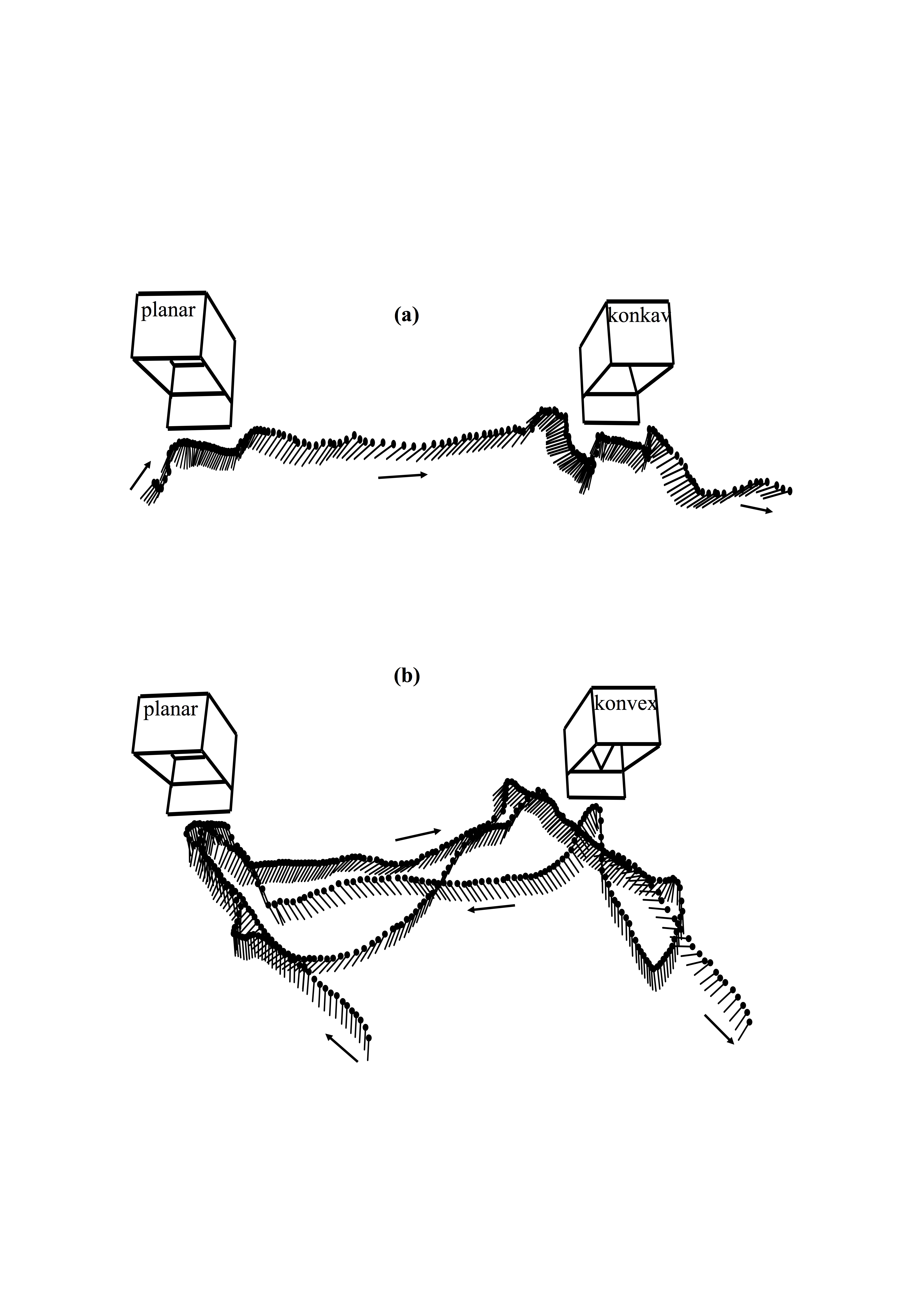
 **Figure A. Flight tracks of individual bees during discrimination tests.** Flight tracks while examining (a) a planar and a concave stimulus and (b) a planar and a convex stimulus. Stimuli are sketched as boxes, as seen from above, labels denote the type of stimulus; dots indicate head-position, black lines correspond to body axis of the bee, arrows indicate direction of flight. Note the reduced translations and change in body axis while inspecting a stimulus, corresponding to a reduction of flight speed.
